# Apelin aggravates inflammatory response and hepatic steatosis in alcohol-associated liver disease via ERK signaling pathway

**DOI:** 10.3389/fphar.2026.1912482

**Published:** 2026-09-30

**Authors:** Hongyan Bian, Aochun Wu, Jinlin Tang, Zhiwei Wang, Jiayi Song, Yi Meng, Xiao Xu, Xinrui Lv

**Affiliations:** 1 Kaifeng Key Laboratory for Infectious Diseases and Biosafety, Kaifeng, China; 2 Oncology Department, The First Affiliated Hospital of Henan University, Kaifeng, Henan, China

**Keywords:** alcoholic liver disease, apelin, APJ, ERK, hepatic Kupffer cell

## Abstract

**Background:**

Though apelin is involved in cardiovascular, metabolic, and homeostasis disorders, its role in alcohol-associated liver disease (ALD) remains unclear. This study aimed to investigate the mechanism of apelin in promoting hepatic inflammation in ALD.

**Methods:**

RAW 264.7 macrophages and a chronic alcohol-fed C57BL/6 mouse model were used. Apelin activated ERK1/2 phosphorylation, and PD98059 pretreatment attenuated apelin-induced MCP-1 and PCNA expression. Inflammatory and proliferative markers (MCP-1, IL-6, PCNA) were assessed by qRT-PCR and western blot. ERK signaling was evaluated using the inhibitor PD98059. Liver pathology was examined by H&E and Oil Red O staining.

**Results:**

Alcohol upregulated apelin expression in RAW 264.7 cells. Apelin-13 increased MCP-1 and PCNA expression in time- and dose-dependent manners. Apelin overexpression enhanced, while siRNA knockdown reduced, inflammatory and proliferative gene expression. Apelin activated ERK1/2 phosphorylation, and PD98059 pretreatment attenuated apelin-induced MCP-1 and PCNA expression. *In vivo*, apelin overexpression exacerbated steatosis, ballooning degeneration, and inflammatory infiltration, with elevated MCP-1, IL-6, and PCNA levels.

**Conclusion:**

Apelin aggravates inflammatory responses and hepatic steatosis in ALD via the ERK signaling pathway, suggesting that the apelin/APJ/p-ERK/inflammation axis may represent a novel therapeutic target for ALD.

## 1 Introduction

Alcohol-associated liver disease (ALD) contributes significantly to the morbidity and mortality among people with liver disorder. However, the main cause behind ALD is alcohol consumption which is involved also in the etiology of fatty liver, alcoholic hepatitis, fibrosis/cirrhosis, and as a result chronic liver disease and hepatocellular carcinoma (Danpanichkul et al., 2026). Although many mechanisms participate in the progression of ALD, including oxidative stress, epigenetic modifications, and derangements in lipid metabolism, inflammation is considered one of the most important factors (Maddur and Flamm, 2026). On the other hand, steatosis is the typical pathological change of ALD as an initial response to heavy drinking which can progress to a severe type of inflammatory injury called steatohepatitis. Meanwhile, tissue maintenance and rapid response to liver injuries are guaranteed by the crucial involvement of hepatic macrophages (Sun et al., 2023). Studies have confirmed that alcohol can induce Kupffer cells (KCs) to release reactive oxygen species (ROS) and proinflammatory cytokines and chemokines that contribute to neutrophil infiltration (Dukić et al., 2023; Suraweera et al., 2015).

Apelin is an endogenous ligand of the specific receptor APJ (angiotensin II receptor-like-1, AT-1), which belongs to the G protein-coupled receptor family. Apelin encodes a secreted 77-amino acid precursor called preproapelin, which is cleaved into a family of apelin fragments (Alfonso, 2026; Davenport et al., 2026; Wyderka et al., 2025). Apelin fragments have various isoforms such as apelin-36, apelin-17, apelin-13 (the most active form), and apelin-12 (Li et al., 2022; Huang et al., 2025). Apelin distributes throughout the body and takes part in cell proliferation, migrations, and apoptosis. As a result, it is involved in the regulation of cardiovascular, metabolic, neurological, hepatic, and diseases, as well as cancer (Agarwala and Sharma, 2025; Respekta et al., 2022; Ivanov et al., 2022; Mehri et al., 2023). Apelin is rarely expressed in primary hepatocytes, normal liver tissues of mouse, and HepG2 cells but is expressed in hepatic non-parenchymal, KCs, stellate, and endothelial cells (Jiang et al., 2025; Zhu et al., 2024; Merens et al., 2025). Recent studies have proposed the role of apelin in progression of wide variety of liver diseases including those mentioned above (Mohamed et al., 2023; Yerra and Drosatos, 2023; Abramczyk et al., 2025). Recent research has demonstrated that Apelin/APJ can inhibit the inflammatory response by down-regulating the nuclear factor κB (NF-κB) pathway and up-regulating the extracellular signal-regulated kinase 1 and 2 (ERK1/2) pathway, and that stable apelin-13 analogues can enhance cellular differentiation and viability while increasing the number of proliferating cells *in vitro* (Wang et al., 2022; Sharma et al., 2025), suggesting that Apelin exert diverse effects depending on tissue type, disease stage, and cellular context. In inflammatory conditions, apelin may participate in either compensatory responses or amplification of inflammatory signaling. Consequently, the potential involvement of apelin in ALD development has emerged as an area of interest. Thus far, we observed that apelin expression significantly increased after inflammatory liver injury. However, its effect in ALD still unclear.

In this study, we investigate the effect of apelin on the expression of proinflammatory cytokines and the enhancement of the inflammatory response in ALD.

## 2 Materials and methods

### 2.1 Animals and experimental design

The 6–8 weeks male mice (SPF, C57BL/6) with 22–24 g were obtained from the Institutional Animal Care and Use Committee of Charles River (Beijing, China; license number: SCXK 2016-0006). All animal experiments performed were conducted in accordance with the US NIH guidelines (Guide for the Care and Use of Laboratory Animals (1985), DHEW Publication No. (NIH) 85–23: Office of Science and Health Reports, DRR/NIH, Bethesda, MD, USA) and the ethical standards of Animal Research Ethics Committee of Henan University (experimental number: HUSOM-2019–135). After acclimation for 1 week, we allocate the mice randomly into control group (n = 6), alcohol-fed group (n = 6), and alcohol-fed–apelin-treated group (n = 6). The control group was fed with the isocaloric dextrose control liquid diet. The other two groups were given unrestricted access to the Lieber–DeCarli alcohol liquid diet with 5% (vol/vol) alcohol. On the first day, the control group and the alcohol-fed group was injected with pCMV plasmid in the tail vein, and the alcohol-fed–apelin-treated group was administered apelin plasmid (15 μg plasmid +200 μL normal saline) via rapid tail vein injection. At the same time on the third day, the corresponding plasmids were administered once again via the tail vein under high pressure using the same dose and method as for the first day. After 10 weeks, mice were euthanized by carbon dioxide asphyxiation (30% chamber volume/min displacement rate), followed immediately by cervical dislocation to confirm death, and then the livers were taken, then stored in 10% formalin for histopathological staining. Or snap-frozen for tests like polymerase chain reaction (PCR) and western blot. Animals were randomly allocated to the experimental groups. No formal blinding procedure was applied during the animal experiments. No formal predefined exclusion criteria were established, and no animals were excluded from the final analysis.

### 2.2 Cell isolation, culture, and treatment

RAW 264.7 cells (obtained from the Cell Bank of the Chinese Academy of Sciences) were cultured in Dulbecco’s Modified Eagle’s Medium (DMEM) supplemented with 10% FBS at 37 °C in a humidified atmosphere with 5% CO_2_. However, the incubation period in a serum-free DMEM lasted 24 h before adding apelin-13(Sigma–Aldrich) or F13A (GL Biochem (Shanghai) Ltd.). In addition, human monocytic THP-1 cells (obtained from the Cell Bank of the Chinese Academy of Sciences) were cultured in RPMI 1640 medium supplemented with 10% FBS and 0.05 mM 2-mercaptoethanol at 37 °C in a humidified atmosphere with 5% CO_2_. The cells were serum-starved for 24 h and then treated with alcohol at concentrations of 0.1 µM and 0.2 µM for 24 h, after which cells were harvested.

### 2.3 RNA preparation and quantitative reverse transcription PCR (qRT-PCR)

While we use TRIzol® reagent (Invitrogen) to isolate RNA, A260/A280 nm absorbance ratio was used to define the purity and concentrations of the substance. Glyceraldehyde-3-phosphate dehydrogenase (GAPDH) gene primers were used as a reference gene in the process of RNA normalization. ABI PRISM 7500 Real-Time PCR System and SYBR Green Master Mix (Applied Biosystems) were used to Each biological sample was analyzed in technical duplicate by qRT-PCR, and the technical replicates were averaged before statistical analysis. Technical replicates were not treated as independent statistical units. Equation R = 2^−ΔΔCT^ was applied to calculate the relative expression level. Nevertheless, we use the following primers: APLN (mouse): 5′-TCT​TGG​CTC​TTC​CCT​CTT​TTC​A-3′ (sense), 5′-GTG​CTG​GAA​TCC​ACT​GGA​GAA-3′ (antisense); GAPDH (mouse): 5′-TGT​GAA​CGG​ATT​TGG​CCG​TA-3′ (sense), 5′-ACT​GTG​CCG​TTG​AAT​TTG​CC-3′ (antisense); IL-6 (mouse), 5′-GTC​CTT​CCT​ACC​CCA​ATT​TCC​A-3′ (sense) and 5′-CGC​ACT​AGG​TTT​GCC​GAG​TA-3′ (antisense); MCP-1 (mouse): 5′-TGG​GCC​TGT​TGT​TCA​CAG​TT-3′ (sense), 5′-ACC​TGC​TGC​TGG​TGA​TTC​TC-3′ (antisense).

### 2.4 Oil Red O staining

After preparing the 7-μm pieces of the frozen liver tissue, we first fixed them with 10% (v/v) formaldehyde for 15 min at normal temperature. Then using double-distilled water (ddH_2_O), slides were washed three times and dried by air after fixing it with formalin. After that, for 10 min, in propylene glycol medium, and at 60 °C, the resulted cryosections were stained with 0.5% Oil Red O, then they washed with 85% propylene glycol. However, the Final stage included counterstaining with hematoxylin, washing with water, mounting with an aqueous solution, and taking photographs for randomly selected liver tissue sections under the microscope.

### 2.5 Western blot

Firstly, SDS–PAGE separated the extracted protein from the cultured cells or liver tissue sample. Then we preserved them on a polyvinylidene difluoride membrane (Millipore, Billerica, MA, USA). The membrane was supplied by 5% nonfat milk in TBST to block nonspecific bindings, and immunoblotted with: 1:300 dilution of rabbit anti-apelin (GeneTex), 1:1000 dilution of rabbit anti-MCP-1 (Abcam), and 1:2500 dilution of rabbit anti-PCNA (Abcam), anti-β-actin, p-ERK/ERK(Cell Signaling Technology, Inc.)and rabbit anti-IgG (Santa Cruz Biotechnology). After incubation with the appropriate secondary antibody, the immunoreactive signal from the antibody–antigen complex was visualized using the Chemiluminescence Plus Western blot analysis kit (Santa Cruz).

### 2.6 Immunofluorescence staining

After fixing RAW 264.7 cells with 4% paraformaldehyde, they were treated with 0.1% Triton X-100, put in an anti-apelin antibody solution, and stained with FITC-conjugated secondary antibody. We use an inverted fluorescence microscope (Leica) to examine the slides. But to observe the nucleus, we used Diamidino-2-phenylindole (DAPI) staining.

### 2.7 Immunohistochemistry

Paraffin-embedded liver sections were deparaffinized, rehydrated, and subjected to antigen retrieval. After blocking, sections were incubated with primary antibodies against Apelin, MCP-1 and PCNA, followed by appropriate secondary antibodies. Immunoreactivity was visualized using DAB, and sections were counterstained with hematoxylin.

### 2.8 Plasmid transfection

Transfection process included apelin expression plasmids from OriGene Technologies Inc. and reagent from LipofectamineTM reagent (Invitrogen). 24 h after the transfection, RAW 264.7 cells were categorized into apelin-treated (100 nM) or apelin-untreated groups. Then, the harvested cells were used for qRT-PCR and western blot analysis.

### 2.9 Phalloidin staining for actin stress fibers

After fixing RAW 264.7 cells with 4% paraformaldehyde, they were treated for 10 min at normal temperature with 0.1% Triton X-100. Later, for 30 min in the dark, we stained the sections with TRITC- phalloidin (Sigma). Inverted fluorescence microscope (Leica) was used to examine the sections. But to observe the nucleus, we used Diamidino-2-phenylindole (DAPI) staining.

### 2.10 Statistical analysis

Data were analyzed using GraphPad Prism 5.0 and are presented as mean ± SD. For the *in vivo* experiments, each individual mouse was considered one biological replicate. For the *in vitro* experiments, each independent experiment was considered one biological replicate. Technical replicates within each independent experiment were averaged and were not treated as independent statistical units. Two-group comparisons were performed using an unpaired two-tailed Student’s t-test. Comparisons among multiple groups were performed using one-way ANOVA followed by Tukey’s *post hoc* multiple-comparison test, where applicable. All statistical tests were two-tailed, and *P* < 0.05 was considered statistically significant.

## 3 Result

### 3.1 Alcohol increased the expression of apelin, APJ, and the related inflammatory genes in RAW 264.7 cells

We investigated whether alcohol influenced macrophages by assessing the protein expression of apelin and APJ, in RAW 264.7 cells using immunofluorescence. According to Figures 1A,B, the fluorescence intensity of staining for apelin and APJ was significantly enhanced when RAW 264.7 cells were treated with alcohol (0.1 μmol/L) for 24 h. To examine how alcohol regulates the expression of inflammation-related genes, we use qRT-PCR on RAW 264.7 cells treated with different concentrations of alcohol for different periods. The results demonstrated that the relative expression of apelin, APJ, MCP-1, and IL-6 mRNA was increased in a time- and dose-dependent manner (Figures 1C–J). These results suggested that an association between the apelin/APJ axis and inflammatory mediators under alcohol stimulation. To further examine whether the alcohol-induced apelin response is reproducible in human monocytic cells, we performed additional qRT-PCR experiments using human THP-1 monocytes. Consistent with the results obtained in RAW 264.7 cells, alcohol treatment (0.1 µM and 0.2 µM for 24 h) significantly upregulated the mRNA expression of APJ and IL-6 in THP-1 cells in a dose-dependent manner (Supplementary Figure S1).

**FIGURE 1:**
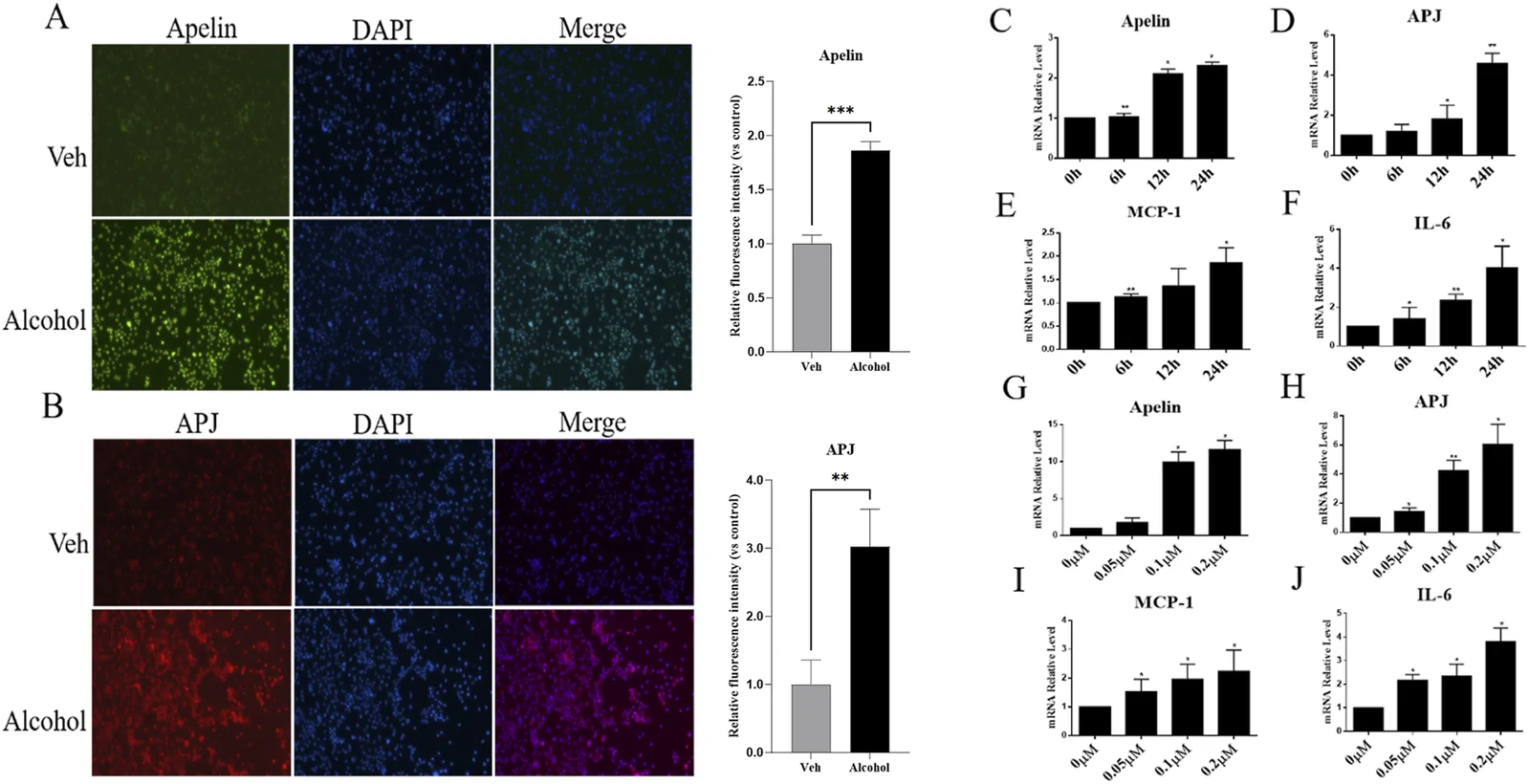
Alcohol induced high level of apelin, APJ, and related inflammatory genes expression. RAW 264.7 cells were exposed to alcohol (0.1 μM/L) for 24 h, fixed, and subjected to immunofluorescence staining **(A,B)**. The RAW 264.7 cells with alcohol (0.1 μM/L) for different time periods **(C–F)** or for 24 h with different alcohol doses **(G–J)**. Total RNA was amplified with PCR after transcription with reverse transcriptase. The internal control was GAPDH. The bars represent mean ± SD (Data are presented as mean ± SD, n = 3 biological replicates, *P < 0.05, **P < 0.01).

### 3.2 Apelin enhanced the mRNA and protein expression of MCP-1 and PCNA in RAW 264.7 cells

With Oil Red O staining, lipid droplets were showed remarkably accumulated in alcohol-treated RAW 264.7 cells (Figures 2A,B). Therefore, when the cells were treated with apelin-13 for various times or different doses, the mRNA and protein expression of MCP-1 and PCNA was increased in a time- and dose-dependent manner (Figures 2C–H). These results demonstrated that apelin participated in the stimulation of inflammation in RAW 264.7 cells.

**FIGURE 2:**
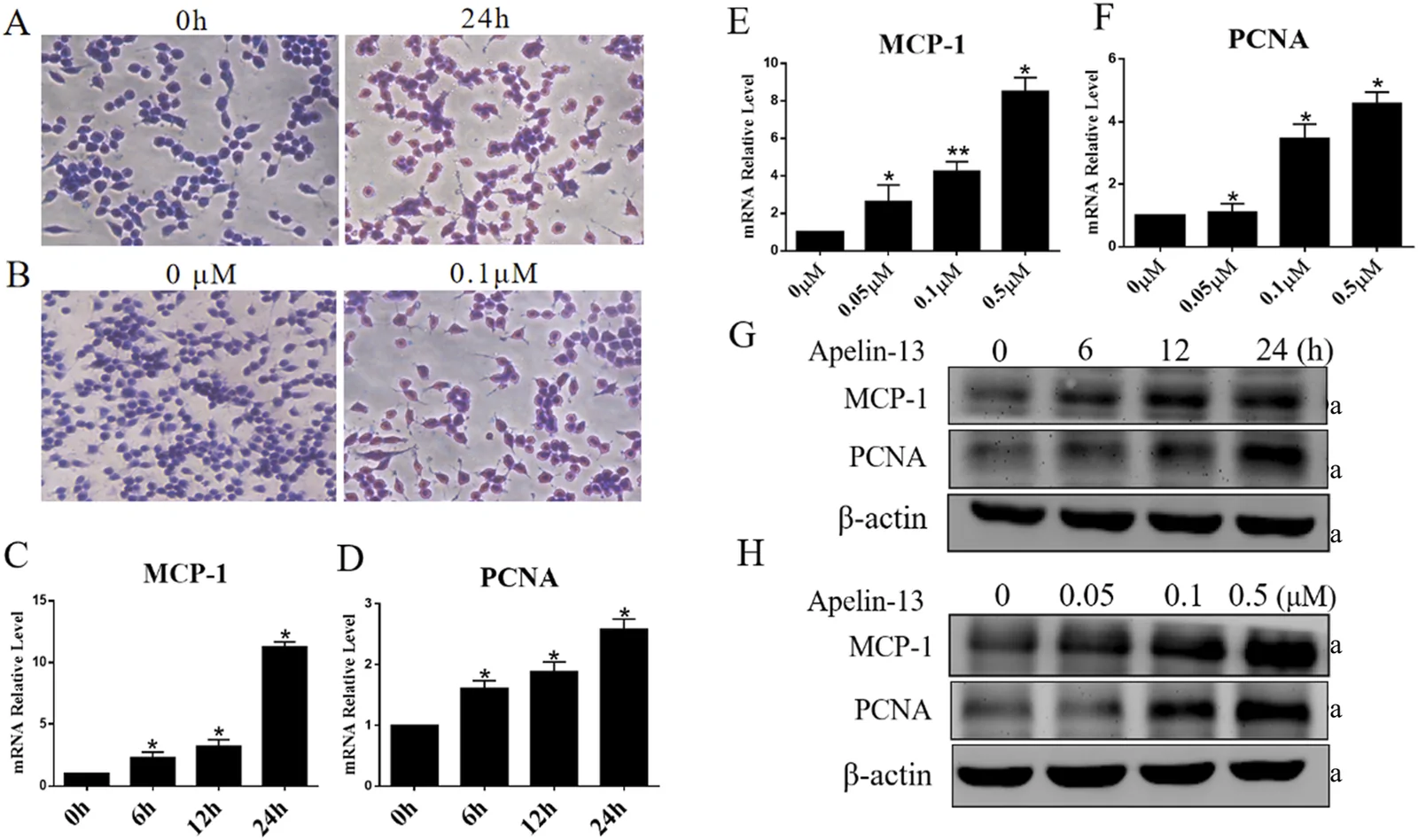
Apelin enhanced the mRNA and protein expression of MCP-1 and PCNA in RAW 264.7 cells. For 24h, RAW 264.7 cells were exposed to 100 nM alcohol, however, lipid accumulation was higher in contrast to the control group **(A,B)**. The cells with apelin-13 for different time periods or for 24 h with different doses of alcohol. Total RNA was amplified with PCR after transcription with reverse transcriptase. The internal control was GAPDH. Bars represent mean ± SD **(C–F)**. In western blot, anti-MCP-1 and anti-PCNA were used as antibodies and β-actin as a loading control **(G,H)**. Experiments were carried out in triplicate (Data are presented as mean ± SD, n = 3 biological replicates,*P < 0.05, **P < 0.01).

### 3.3 Apelin played a positive regulatory role in proinflammatory and proliferative genes expression in RAW 264.7 cells

As a further evaluation of the importance of apelin in regulating the expression of inflammation-related genes, RAW 264.7 cells were infected with apelin expression plasmids or transfected with apelin siRNA to overexpress or knockdown endogenous apelin expression, respectively. The qRT-PCR, western blot, and phalloidin staining results showed that apelin overexpression markedly increased the mRNA and protein expression of MCP-1, IL-6, PCNA (Figures 3A–C,G), and F-action expression (Figure 4A). Conversely, the knockdown of endogenous apelin expression with apelin siRNA showed that the mRNA and protein expression of MCP-1, IL-6, and PCNA clearly decreased (Figures 3D–F,H), along with the expression of F-action (Figure 4B). These results suggested that apelin contributes to the regulation of inflammatory and proliferative responses in RAW 264.7 cells.

**FIGURE 3:**
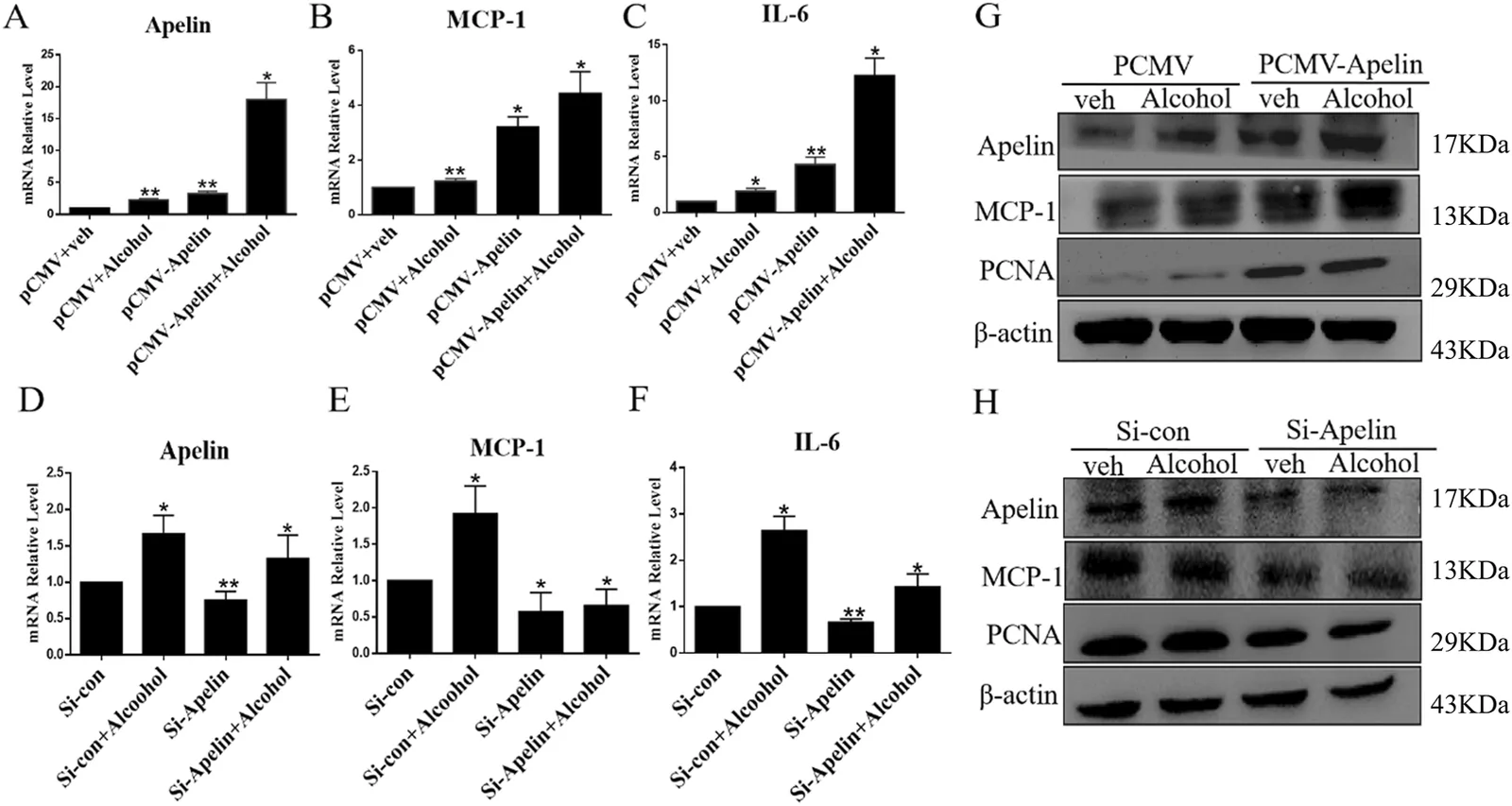
Overexpression or knockdown of apelin regulated MCP-1 and PCNA mRNA and protein levels in RAW 264.7 cells. Either with PCMV-apelin or si-apelin, RAW 264.7 cells were transfected for 24 h and then exposed to alcohol (100 nM) for 24 h. Harvested cells were analyzed by qRT-PCR (3A–F) and western blot (3G–H). Total RNA was amplified with PCR after transcription with reverse transcriptase. The internal control was GAPDH. Anti-MCP-1 and anti-PCNA were used as antibodies and β-actin as a loading control to analyze. Crude proteins extracted from treated cells with western blot. Experiments were carried out in triplicate (Data are presented as mean ± SD, n = 3 biological replicates,*P < 0.05, **P < 0.01).

**FIGURE 4:**
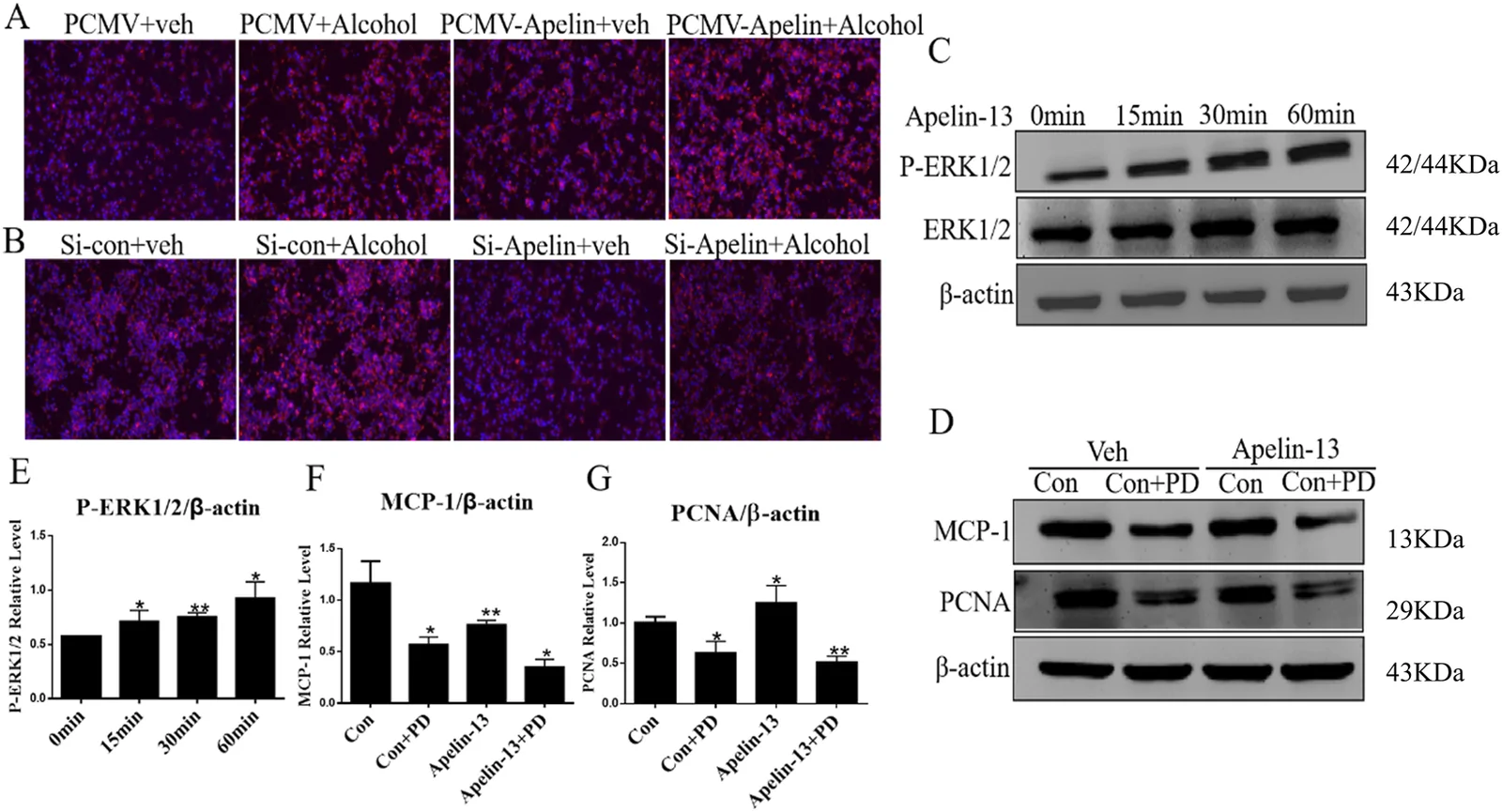
APJ blockade attenuates apelin-induced ERK activation and downstream inflammatory responses. Either with PCMV-apelin or si-apelin, RAW 264.7 cells were transfected for 24 h and then exposed to alcohol (100 nM) for 24 h. The slides were fixed and stained with TRITC-phalloidin to visualize F-actin **(A,B)**. The cells were exposed to 100 nM apelin-13. To analyse total cell lysates, western blot was used with p-ERK and ERK antibodies and β-actin as endogenous control **(C)**. For 2 h, RAW 264.7 cells were incubated with PD98059 (25 μM/L), then for 24 h with or without apelin-13 (100 nM). Subsequently, the cells were collected to conduct western blot with anti-MCP-1 or anti-PCNA antibodies and β-actin as a loading control **(D)**. The band intensities were quantized using the Photoshop Image Tanon software **(E–G)** (Data are presented as mean ± SD, n = 3 biological replicates, *P < 0.05,**P < 0.01).

### 3.4 APJ blockade attenuates apelin-induced ERK activation and downstream inflammatory responses

We investigate the change in the ERK signaling pathway by using a western blot assay. After 15 min of adding apelin-13, the result showed a higher level of p-ERK1/2, which lasted for 1 hour (Figures 4C,E). Another, ERK inhibitor, PD98059, was added 2 h before adding apelin-13. As a result, inhibition of ERK subsequently decreased the expression of MCP-1 and PCNA. The previous test demonstrated that the role of the ERK pathway in the regulation of apelin, which means the expression of proinflammatory and pro-proliferative genes in RAW 264.7 cells. To determine whether the activation of ERK signaling by apelin was dependent on the apelin receptor APJ, RAW 264.7 cells were pretreated with the APJ antagonist F13A before apelin-13 stimulation. Compared with apelin-13 treatment alone, F13A markedly reduced apelin-induced ERK1/2 phosphorylation, accompanied by decreased MCP-1 and PCNA expression (Supplementary Figure S2). These findings indicate that APJ functions upstream of ERK signaling and mediates apelin-induced inflammatory and proliferative responses.

### 3.5 Apelin regulates hepatic metabolic and oxidative stress-related responses in ALD mice

Hematoxylin and eosin (H&E) staining revealed that the liver tissue structure of the control group was clear and that the liver cords were neatly arranged, with no damage in the central vein or the portal area. In contrast, hepatocellular steatosis was around the central veins of hepatic lobules in the alcohol-fed group. In the alcohol-fed–apelin-treated group, foamy fatty lesions were observed, and large adipose vacuoles were apparent in the cytoplasm (Figure 5A). Oil Red O staining revealed remarkable accumulation of droplets in the liver tissues of the alcohol-fed–apelin-treated group in contrast to the model group (Figure 5B). These results demonstrated that apelin overexpression aggravated alcohol-associated hepatic steatosis and inflammatory pathological changes. In particular, Cyp2e1 and Mas1 expression was modified following apelin intervention, suggesting that apelin may influence alcohol-induced oxidative injury (Supplementary Figure S3).

**FIGURE 5:**
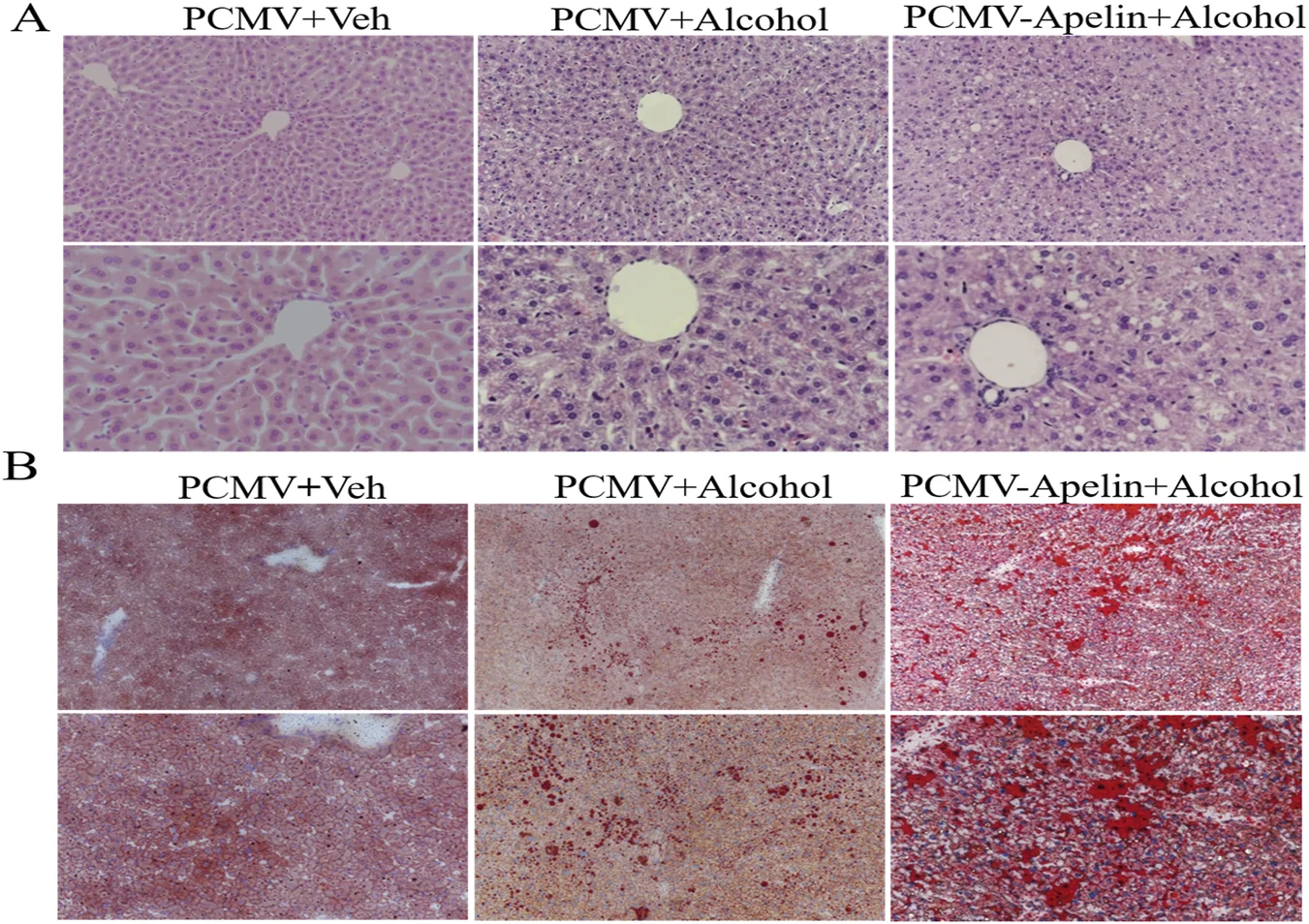
Apelin regulates hepatic metabolic and oxidative stress-related responses in ALD mice. Liver histology (H&E staining, magnification: upper ×200, lower ×400) **(A)**. Liver Oil Red O staining (magnification: upper ×200, lower ×400) **(B)** (n = 6 mice per group).

### 3.6 Apelin induced the upregulation of the expression of proinflammatory and proliferative genes in the liver tissues in the apelin-treated ALD mouse model

The apelin-treated specimen of liver tissue was analyzed with immunohistochemistry (IHC). However, it showed that the expression of apelin and inflammatory- and proliferation-related genes, such as MCP-1 and PCNA, were clearly upregulated in these specimens in contrast to alcohol-fed group (Figures 6A–C). The qRT-PCR and western blot results demonstrated that the overexpression of apelin lead to over-expression of MCP-1, IL-6, and PCNA in the alcohol-fed–apelin-treated group (Figures 6D–F). As a result, apelin involved in the hepatic inflammation and proliferation in the ALD mouse model (Figure 7).

**FIGURE 6:**
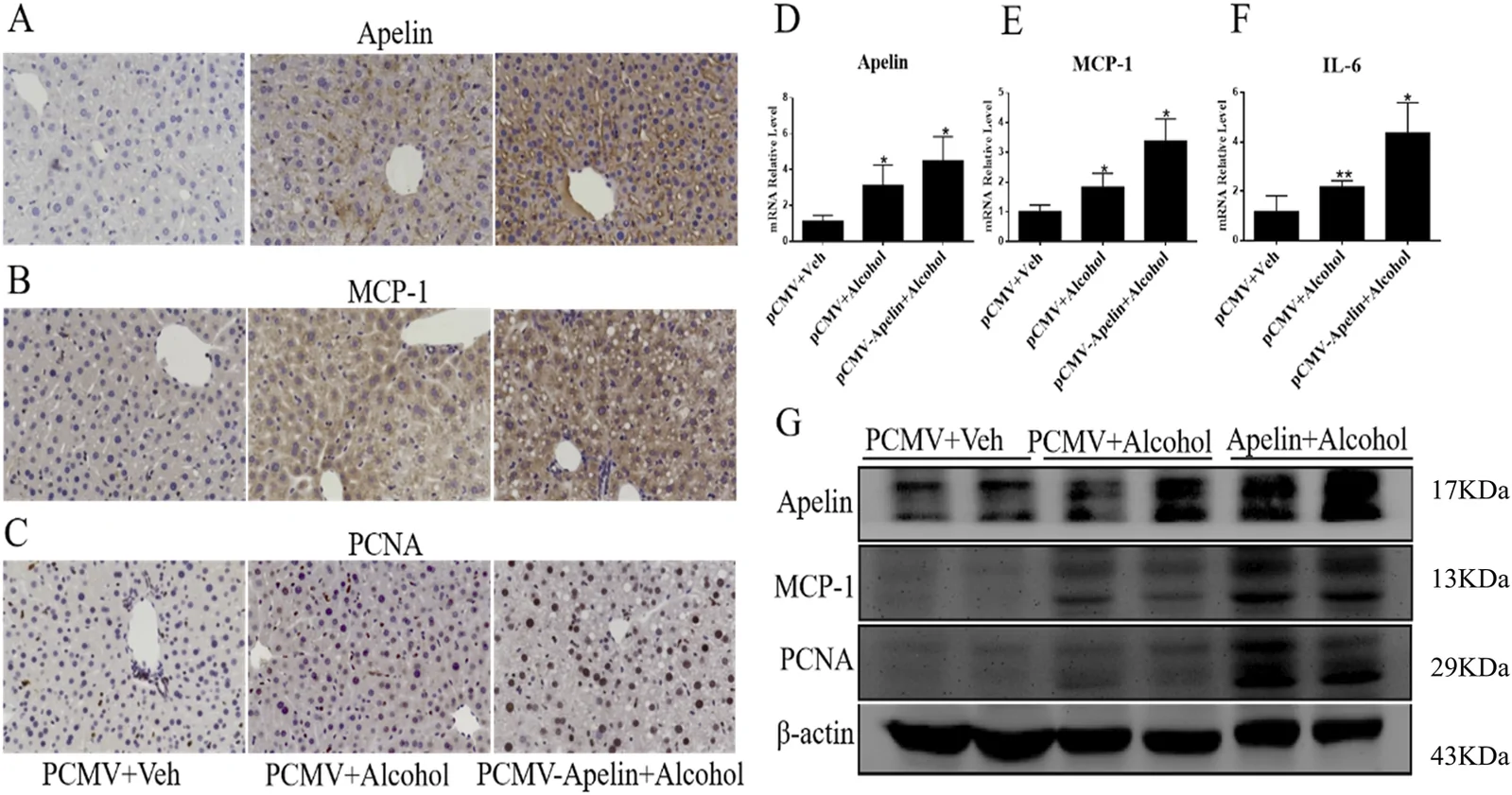
Apelin induced the upregulation of proinflammatory and proliferative genes in the liver tissues of the ALD mouse model. The expression of MCP-1 and PCNA in the liver tissues of different groups of mice, as shown by IHC analysis (A–C). Using qRT-PCR, the mRNA expression of apelin, MCP-1, and IL-6 in the liver tissues of the mice (D–F). Western blot with anti-MCP-1 and anti-PCNA antibodies and β-actin a loading control was used to investigate the protein expression(G) (Data are presented as mean ± SD, n = 6 mice per group, *P < 0.05,**P < 0.01).

**FIGURE 7:**
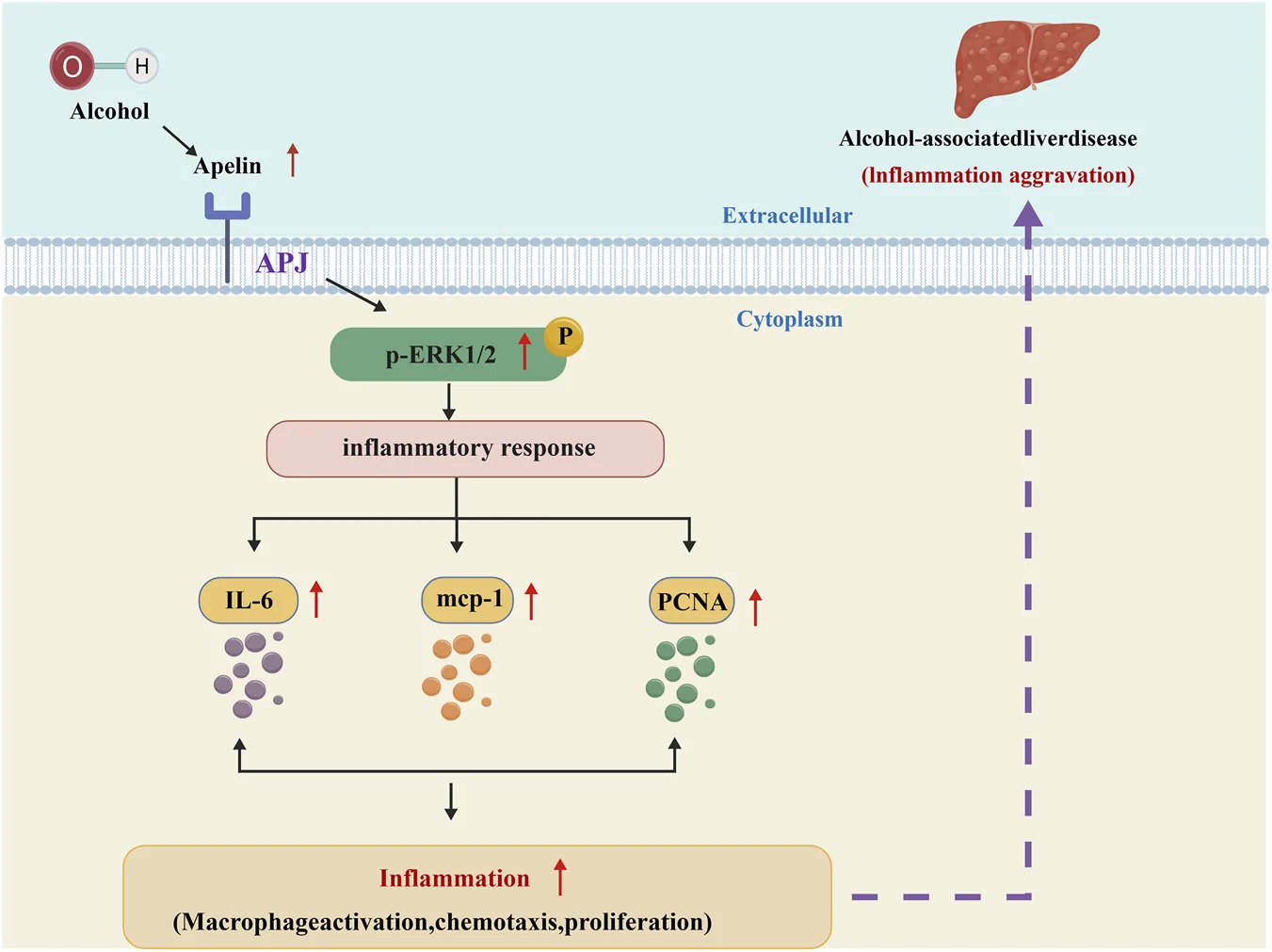
Mechanistic model: Proposed mechanism of apelin in promoting hepatic inflammation and proliferation in alcohol-associated liver disease.

## 4 Discussion

Long-term intake of alcohol can damage liver function and eventually lead to ALD. ALD is an inflammatory reaction directly or indirectly induced by alcohol and its derivatives during metabolism (Wang et al., 2020). During the pathogenesis of ALD, steatosis cab transforms to steatohepatitis due to excessive or chronic inflammatory reactions, even causing the death of large numbers of liver cells, indicating a more severe type of inflammatory liver injury (Shen et al., 2025). Specialized hepatic macrophages (KCs) critically participate in the development of ALD. The activation of KCs and the recruitment of liver neutrophils may lead to inflammatory responses in the liver and an increase in the production of inflammatory mediators (Gao et al., 2024). Here, we provide evidence that elevated apelin expression is associated with enhanced inflammatory responses and steatotic changes in ALD models. To further characterize the steatotic phenotype observed *in vivo*, we examined Cyp2e1, a key enzyme in alcohol metabolism and oxidative stress. Cyp2e1 expression was modulated by apelin overexpression, suggesting that apelin may influence alcohol-induced oxidative injury. The precise lipid metabolic pathways underlying this phenotype have yet to be fully characterized, and further studies are needed to clarify whether apelin drives steatosis through direct metabolic regulation or indirectly via inflammatory amplification. Nonetheless, our findings provide an initial molecular link between apelin signaling and the steatotic phenotype in ALD.

In many organs, apelin takes part in the physiological and pathological activity, including cardiovascular function; metabolic balance; neurological function; and cell proliferation, apoptosis, or inflammation. It can be separated into several active peptide forms, such as apelin-13, apelin-16, apelin-17, and apelin-36. Each isoform is capable of the potent activation of APJ, although apelin-13 exerted the strongest activity (Respekta et al., 2022; Wen et al., 2023). The study has shown that although apelin expression is distributed in the tissue of various peripheral organs, it is low in healthy liver tissue. Previous studies have reported that in patients with liver cirrhosis, the concentration of apelin either in the blood or in the affected liver tissue was significantly higher than in the control groups (Lim et al., 2016). Apelin is involved in the development of inflammation, and the addition of LPS, IL-6, or TNF-α to HepG2 or LX-2 cells increases the expression of apelin (Melgar-Lesmes et al., 2011). Although apelin has been reported to exert protective effects in several metabolic and cardiovascular diseases, our findings suggest that its function may be highly dependent on pathological context. Under chronic alcohol exposure, increased apelin signaling may contribute to inflammatory amplification through apelin/APJ/p-ERK/inflammation axis activation.

The most prominent pathological feature of ALD is fatty liver formation and inflammatory hyperplasia. Studies have shown that alcohol could activate hepatic macrophages to produce ROS and proinflammatory cytokines or chemokines, such as IL-6, IL-8, IL-1, TNF-α, and MCP-1, thereby promoting the production of liver inflammation (Sarode et al., 2026; Guan et al., 2024; Ge et al., 2024). The level of proinflammatory cytokines was increased in alcohol-treated RAW 264.7 cells, and the expression of apelin and APJ was also significantly increased (Figures 1A–G). Previously, some proinflammatory cytokines were shown to increase the expression of apelin, and Apelin can activate the expression of inflammatory cytokines such as TNF-α, IL-1β, IL-10, and MCP-1 in BV2 microglia via the PI-3K/Akt and MEK/ERK pathwayss (Chen et al., 2015). In our *in vitro* experiments, apelin-13 increased the mRNA and protein expression of MCP-1 and PCNA in RAW 264.7 cells in a time- and dose-dependent manner (Figures 2C–H).

On the other hand, the RAW 264.7 cells were transfected with apelin expression plasmids or apelin siRNA to overexpress or knockdown endogenous apelin expression, respectively. The overexpression of apelin significantly increased the mRNA and protein expression of MCP-1, IL-6, and PCNA (Figures 3A–C,G). Conversely, the knockdown of endogenous apelin expression decreased the mRNA and protein expression of MCP-1, IL-6, and PCNA (Figures 3D–F,H). Our above *in vitro* results were consistent with those obtained *in vivo*. Overall, it is clear that apelin takes part the hepatic inflammation and proliferation in ALD of mice. Moreover, phalloidin staining demonstrated the appearance of brighter and more condensed F-actin stress fibers surrounding the nucleus of RAW 264.7 cells overexpressing apelin or treated with alcohol (Figures 4A,B). These results indicated that apelin could alter actin cytoskeletal organization and promote migration in RAW 264.7 cells.

Apelin binding to APJ activates multiple downstream signaling pathways, including G protein–mediated PI3K/Akt and MAPK/ERK cascades, which collectively contribute to phosphorylation of proteins involved in cell proliferation and migration (Naldi et al., 2025). Furthermore, in H9c2 cardiomyoblast cells, apelin-13 has been demonstrated to promote cell proliferation by triggering extracellular signal-regulated kinase 1/2 (ERK1/2) and protein kinase B (Akt) phosphorylation (Yin et al., 2018). After the addition of apelin-13 to RAW 264.7 cells, we found a higher p-ERK1/2 level within 15 min lasted for 1 h. On the other hand, 2 h before apelin-13 treatment, we add RAW 264.7 cells with the ERK inhibitor PD98059. However, the results showed that PD98059 significantly decreased the expression of MCP-1, and PCNA in RAW 264.7 cells as a result of apelin inhibition (Figures 4C–G). These findings indicate that ERK signaling mediates, at least partially, apelin-induced inflammatory and proliferative responses.

In summary, moderate levels of apelin and alcohol led to significant hepatic inflammation and steatosis. Our study has explored the pivotal role of apelin hepatic disease such as ALD. In our study, the level of proinflammatory cytokines was increased after apelin stimulation in alcohol-treated RAW 264.7 cells and ALD mouse model. Based on our findings, we propose a mechanistic model (Figure 7) illustrating how apelin promotes hepatic inflammation and proliferation in ALD. Furthermore, our findings suggest that apelin may contribute to inflammatory and proliferative responses in macrophages, at least in part, through ERK signaling. This raises the possibility that the apelin/APJ/p-ERK/inflammation axis could represent a potential therapeutic target for ALD. However, given the well-established protective roles of apelin in cardiovascular and metabolic homeostasis, systemic inhibition of this pathway might carry inherent safety risks. These challenges might be addressed through liver-targeted delivery strategies, time-limited administration during the acute inflammatory phase, or the development of biased ligands that could selectively disrupt pro-inflammatory signaling while preserving beneficial downstream pathways. Such approaches may help to dissociate therapeutic efficacy from off-target toxicity and merit further exploration.

## Data Availability

The original contributions presented in the study are included in the article/Supplementary Material, further inquiries can be directed to the corresponding authors.
